# Structural Plasticity of the Selectivity Filter in Cation Channels

**DOI:** 10.3389/fphys.2021.792958

**Published:** 2021-12-07

**Authors:** Kitty Hendriks, Carl Öster, Adam Lange

**Affiliations:** ^1^Department of Molecular Biophysics, Leibniz-Forschungsinstitut für Molekulare Pharmakologie, Berlin, Germany; ^2^Institut für Biologie, Humboldt-Universität zu Berlin, Berlin, Germany

**Keywords:** protein dynamics, asymmetry, ion channel, channel gating, ion conduction

## Abstract

Ion channels allow for the passage of ions across biological membranes, which is essential for the functioning of a cell. In pore loop channels the selectivity filter (SF) is a conserved sequence that forms a constriction with multiple ion binding sites. It is becoming increasingly clear that there are several conformations and dynamic states of the SF in cation channels. Here we outline specific modes of structural plasticity observed in the SFs of various pore loop channels: disorder, asymmetry, and collapse. We summarize the multiple atomic structures with varying SF conformations as well as asymmetric and more dynamic states that were discovered recently using structural biology, spectroscopic, and computational methods. Overall, we discuss here that structural plasticity within the SF is a key molecular determinant of ion channel gating behavior.

## Introduction

**FIGURE 1:**
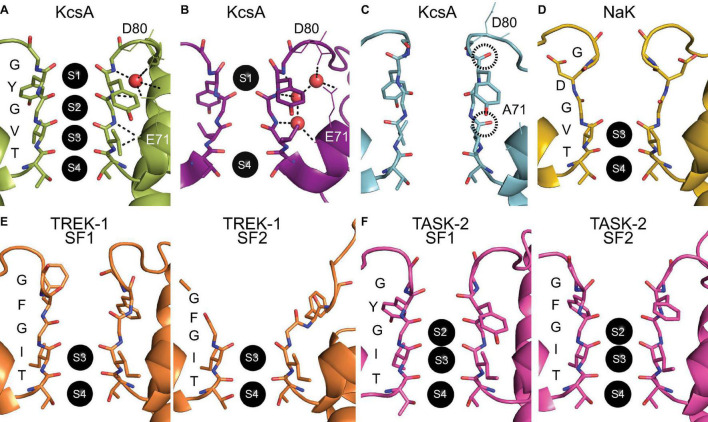
Atomic structures of selectivity filter conformations. **(A)** The conductive conformation of the SF of KcsA (PDB ID: 1K4C) with two opposing subunits depicted and with the sequence and ion binding sites indicated. **(B)** The collapsed SF conformation of KcsA under low potassium conditions (PDB ID: 1K4D). **(C)** The flipped SF conformation of the E71A KcsA mutant (PDB ID: 2ATK) with the reoriented backbone carbonyl groups of the SF circled. Residues D80 and E71 are indicated and depicted as lines. The hydrogen bond network is symbolized with black dashed lines and water molecules are depicted as red spheres in one of the two opposing subunits. **(D)** The crystal conformation of the SF of NaK (PDB ID: 3E86). **(E)** The asymmetric SF conformations of TREK-1 under low potassium conditions (PDB ID: 6W7C) with the opposing subunit pairs of SF1 and SF2 shown. **(F)** The asymmetric SF conformations of TASK-2 at pH 6.5 (PDB ID: 6WLV) with the opposing subunit pairs of SF1 and SF2 shown.

The ability of ion channels to permit ion flux across biological membranes is essential for the functioning of a cell. There are a wide array of diseases associated with ion channel dysfunction that are collectively termed channelopathies including epilepsy, cardiac arrhythmia, deafness, asthma, and cancer (Kim, 2014). Pore loop channels are a family of tetrameric ion channels including potassium-, calcium-, and sodium-selective channels that all share a pore domain architecture. The pore domain consists of two transmembrane helices connected via a reentrant loop that forms the selectivity filter (SF) and a stabilizing pore helix (MacKinnon, 1995). For potassium-selective channels the SF is made from the conserved sequence TVGYG (Heginbotham et al., 1994). The backbone carbonyl oxygens plus the hydroxyl group of the threonine form four ion binding sites, called S1 to S4 from the extracellular side (see Figure 1A), and they perfectly mimic the hydration shell of potassium ions (Liu et al., 2012). There is an additional S0 site above the SF sequence with less precise ion coordination. The non-selective channel NaK has a similar SF sequence of TVGDG but forms only two ion binding sites that are equivalent to S3 and S4 (Shi et al., 2006). It is a model system for other non-selective pore loop channels, such as the hyper-polarization cyclic nucleotide-gated (HCN) channel (Lee and MacKinnon, 2017) and cyclic nucleotide-gated (CNG) channels (Li et al., 2017; Xue et al., 2021). Within the pore loop family there are several channels with a non-traditional SF sequence, such as calcium or sodium channels (Payandeh et al., 2011; Naylor et al., 2016). Here we will focus only on the potassium-selective and non-selective cation channels with traditional SF sequences.

The SF can act as a gate and modulate the electrophysiological behavior of the ion channel. One regulatory mechanism is C-type inactivation that was first discovered in voltage-gated potassium channels, where prolonged activation of the channel by an external stimulus leads to a non-conductive conformation of the SF. In the model potassium channel KcsA high external potassium concentrations are able to slow C-type inactivation, which highlights the link between the SF and C-type inactivation (Cuello et al., 2010).

In recent years there has been a strong increase in research demonstrating the dynamic nature of the SF in various channels. Commonly used structural biology techniques, such as X-ray crystallography or cryogenic electron microscopy (cryo-EM), lead to static snapshots of biological processes where the dynamics can easily be overlooked. Molecular dynamics (MD) simulations complement experimental approaches by obtaining information on the dynamics of previously determined structures (Li et al., 2018; Kopec et al., 2019; Saponaro et al., 2021). However, MD simulations are still limited in terms of the timescale that can be sampled and sometimes suffer from the inaccuracy of force field parameters. Solid-state nuclear magnetic resonance (ssNMR) spectroscopy is a technique that is able to detect and quantify the dynamics of membrane proteins, all while maintaining native-like conditions of a lipid bilayer environment at physiological conditions and temperatures (Wylie et al., 2014; Van Der Cruijsen et al., 2017; Mandala et al., 2018; Jekhmane et al., 2019). Combining various types of experimental data with MD simulations allows for a more complete investigation of biological systems, including the dynamics.

We will discuss several modes of structural plasticity for the SF region of potassium-selective and non-selective cation channels; specifically collapse, asymmetry and disorder. These discoveries highlight recent developments in the field and indicate the importance of SF plasticity in pore loop channel function.

## Selectivity Filter Collapse

The first glimpse of structural plasticity of the SF came from crystal structures of the conductive and collapsed conformations of KcsA with high and low potassium concentrations, respectively (Doyle et al., 1998; Zhou et al., 2001). The conformations described only concern the SF, as the activation gate of KcsA is in a closed state for both structures. The conductive SF conformation has the canonical four ion binding sites, whereas the collapsed conformation is constricted with only the S1 and S4 binding sites remaining (see Figure 1B). The constriction at the S2 site is caused by a reorientation of the glycine residue in the middle of the SF and this is stabilized through interactions with three water molecules behind the SF (Zhou et al., 2001). The SF conformational change is correlated to the potassium concentration as was also shown using NMR titration experiments (Chill et al., 2006; Bhate et al., 2010). The pore helix region surrounding the SF influences the transition between the conductive and collapsed SF state as demonstrated through a combination of point mutation experiments and MD simulations (Cordero-Morales et al., 2007). Solid-state NMR studies on KcsA showed allosteric regulation of the SF conformation by the activation gate, a pH sensor at the C-terminal end of the protein (Wylie et al., 2014; Xu et al., 2019). This allosteric control of the collapsed SF conformation was also observed in recent microsecond-scale MD simulations (Li et al., 2018), which clearly revealed that the SF of KcsA acts as another gate and plays an important role for ion channel functioning.

Mutations of E71 in the pore helix of KcsA change the equilibrium between the conductive and collapsed SF conformations. The E71A mutant was crystallized in two distinct conformations, one similar to wild-type KcsA and one with several flipped residues (Cordero-Morales et al., 2006). The flipped conformation shows W67 in the pore helix and D80 as well as V76 in the SF with a reoriented sidechain or carbonyl group (see Figure 1C). This flipped conformation was found again when the E71A mutant was crystallized under sodium conditions (Cheng et al., 2011). The flipped conformation was suggested to be responsible for the increased sodium conduction observed in the E71A mutant and therefore gives some insight into the principles behind selective ion conduction.

A recent study using MD simulations of a homology model of the Shaker channel also displayed a stable constricted SF conformation that was allosterically regulated (Li et al., 2021b). This conformation was stabilized by only one water molecule behind the SF and a hydrogen bond ring of donor-acceptor pairs at the bottom of the SF is in the opposite direction compared to KcsA, because the SF valine residue is in a flipped orientation. MD simulations of the KcsA mutant E71V revealed the same constricted conformation as for the Shaker channel, which has a valine residue at the position equivalent to E71 in KcsA (Li et al., 2021b).

## Selectivity Filter Asymmetry

All SF conformations described so far assume fourfold symmetry across the tetramer assembly, but this is not necessarily the case. A functional example of an asymmetric SF conformation was found in the non-selective channel NaK with the unique SF sequence TVGDGN. Using ssNMR we discovered that the channel adopts two distinct SF conformations, stabilized by either potassium or sodium ions (Shi et al., 2018). This directly contradicted the previously reported identical structures of NaK that had been crystallized in the presence of potassium or sodium ions (see Figure 1D; Shi et al., 2006). One of the discovered SF conformations corresponds to the crystal structure of NaK, whereas the other conformation consists of a backbone carbonyl flipped conformation that distorts the SF. This flipped conformation was shown in MD simulations to be essential in creating an asymmetric pore, which has a mixture of subunits in the crystal and flipped conformation, and allows for efficient sodium permeation (Shi et al., 2018). The predicted sodium permeation pathway contained a novel side-entry ion binding site, which is the result of the sidechain reorientation of the SF residue D66. A recent crystal structure of NaK revealed dual conformations of two residues in the SF, among them D66, and confirmed this side-entry ion binding site (Roy et al., 2021). In contrast, for potassium permeation the symmetric pore with all subunits in the crystal conformation is required in NaK (Shi et al., 2018). A recent study characterized the structural plasticity of NaK on multiple timescales using solution NMR (Lewis et al., 2021). The results from this study confirmed the ion dependence of the SF, which was even shown to continue along an allosteric pathway that couples the SF conformation to the lower region of the channel.

Since then, SF asymmetry has been observed in other pore loop channels. A subset of pore loop channels that lend themselves especially well for asymmetric SF configurations are K_2*P*_ channels. These channels consist of two dimers that form a pseudo-tetramer that can have different SF sequences, often with a tyrosine to phenylalanine substitution [T(V/I)GFG] in one or both of the SF sequences. The primary gating of K_2*P*_ channels occurs at the SF (Schewe et al., 2016). In a series of recent cryo-EM structures, the SF of the K_2*P*_ channel TREK-1 undergoes changes at low potassium concentrations (Lolicato et al., 2020). The SF1 of opposing subunits showed a pinching movement whereas the other SF2 pair of opposing subunits showed a dilation, disrupting the S1 and S2 ion binding sites (see Figure 1E). These changes highlight the dynamic nature of the inactivated state of the channel and were suppressed by the addition of a small molecule activator (Lolicato et al., 2020). Similarly, a recent cryo-EM structure of another K_2*P*_ channel, TASK-2, revealed that the closed channel is asymmetric instead of the open pseudo-fourfold symmetric channel (Li et al., 2020). There is an asymmetric expansion at the S1 site and strikingly the S0 site has a constriction for the opposing SF1 subunits, but an expansion for the SF2 subunit pair (see Figure 1F). Overall, this deformation of the SF in TASK-2 is not as pronounced as that observed for TREK-1. However, it is likely that other K_2*P*_ channels have similar asymmetric conformations of the SF as closed states of the channel that disrupt ion binding sites.

Recent work on an inactivated mutant of the potassium-selective Shaker channel showed a similar SF conformation to the closed TASK-2 channel (Li et al., 2021b). MD simulations revealed the SF conformation of the W434F mutant (equivalent to W67 in KcsA) where the aspartate residue above the SF has flipped outward and the SF is stabilized by the sidechain of the neighboring residue in an asymmetric constriction of the S0 site. The constriction at the top of the SF does not seem to be allosterically linked to the activation gate, in contrast to the constriction at the S2 ion binding site seen in KcsA. The similarity between the asymmetric conformations of different pore loop channels, K2P and Shaker, points toward a potentially conserved mechanism.

The human ether-a-go-go related gene (hERG) channel is a pore loop channel that functions as a fast-inactivating potassium-selective channel, which has a phenylalanine instead of a tyrosine in its SF sequence (SVGFG). The cryo-EM structure of the open state of the channel revealed that the sidechain of the phenylalanine was slightly shifted compared to other potassium-selective channels, and this was proposed to be linked to the fast inactivation of the hERG channel (Wang and MacKinnon, 2017). A recent study of the hERG channel using MD simulations resulted in asymmetric SF constrictions for which no ion conduction was observed (Li et al., 2021a). The phenylalanine sidechains of two opposing subunits reorient and form stabilizing hydrogen bonds behind the other subunit. This reorientation constricts the conduction pathway at the S2 site, which is not constricted in the other pair of opposing subunits. Multiple mutants were analyzed and a correlation was found between the level of inactivation and the stabilization of the asymmetric SF conformation.

## Selectivity Filter Disorder

The previously discussed non-selective channel NaK can be made potassium-selective by two point mutations in the SF sequence from TVGDGN to TVGYGD or TVGFGD (Alam and Jiang, 2009; Sauer et al., 2011). We have shown using ssNMR that these potassium-selective mutants lose their SF stability under sodium conditions and become disordered without stabilizing potassium ions (Öster et al., 2019; Hendriks et al., 2021). The loss of SF stability strongly influences the hydrogen bonding network behind the SF and through this network impacts the pore helix (Hendriks et al., 2021). Reintroduction of potassium ions allowed for the stabilization of the SF into the canonical four ion binding sites conformation. This finding shows the intrinsic instability of the potassium-selective SF and highlights the interplay between the SF and the permeant ions. It also highlights that a stable closed SF conformation, as found for the collapsed conformation in KcsA, is not available to all pore loop channels.

Another example of SF disorder comes from recent work using FRET (Förster resonance energy transfer) in the potassium-selective channel KirBac1.1. A decrease in the concentration of potassium ions changes the SF from a rigid high-FRET state to a more dilated and dynamic state with medium and low-FRET signals (Wang et al., 2019). This increase in disorder again shows that interactions with the permeant ion are essential for maintaining the integrity of the SF conductive conformation.

KcsA displays three different gating modes within the wild-type channel which can be mimicked by a set of three mutants for E71 (Chakrapani et al., 2011). Solid-state NMR investigations of these mutants pointed toward the water molecules behind the SF stabilizing different conformational landscapes (Jekhmane et al., 2019). The dynamics of the SF clearly have a great effect on the electrophysiological behavior of the channel.

Microsecond-timescale MD simulations of the hERG channel show SF instability (Miranda et al., 2020). This is in contrast to the stable asymmetric conformation of hERG discussed previously (Li et al., 2021a). Two distinct metastable states were found for hERG with transitions between the two states on a low-microsecond timescale, indicating the overall instability of the SF (Miranda et al., 2020). Both states were defined as non-conducting and are characterized by asymmetric SF conformations, where the distances between SF residues of opposing subunit pairs differ significantly. The most populated state shows a constricted pore for one SF pair, whilst the other pair remains structurally similar to the original cryo-EM structure. Importantly, the sidechain of the phenylalanine residue also displays large fluctuations in this state. The less populated metastable SF state is distinctive in the fact that the sidechain of the phenylalanine of one subunit blocks the conduction pathway of the channel. This study describes a SF that is both asymmetric as well as disordered, more research is needed to determine if this is linked to the fast inactivation of the channel.

## Discussion

**FIGURE 2:**
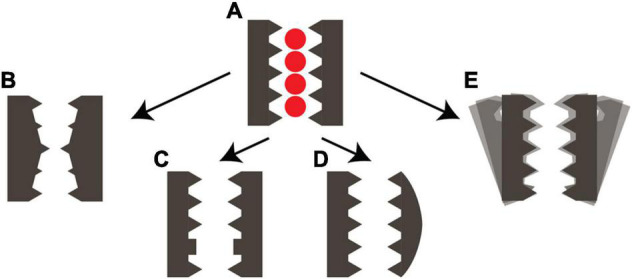
Structural plasticity of the selectivity filter of cation channels. **(A)** Schematic representation of the conductive SF conformation with four ion binding sites. Arrows indicate the conversion into **(B)** a stable collapsed conformation as observed in KcsA (Zhou et al., 2001) or Shaker (Li et al., 2021b). **(C)** A carbonyl flipped conformation as observed in KcsA E71A (Cordero-Morales et al., 2006; Cheng et al., 2011) or NaK (Roy et al., 2021). **(D)** an asymmetric conformation as observed in NaK (Shi et al., 2018) or TREK-1 (Lolicato et al., 2020) or TASK-1 (Li et al., 2020). **(E)** An unstable disordered SF conformation as observed in hERG (Miranda et al., 2020) or the potassium-selective mutants of NaK in the presence of sodium (Hendriks et al., 2021).

It has become increasingly clear in recent years that structural data alone does not give enough information to describe ion channel functioning. Recent research has shown that a lack of dynamic information has limited the mechanistic understanding of ion channels. We have summarized all examples of structural plasticity of the SF in the pore loop channel family, focusing on the potassium- and non-selective cation channels. The SF structures include various constricted, stable conformations as well as asymmetric or even disordered states (see Figure 2). Recently, the TRPV1 and the HCN4 pore loop channels were discovered to have more subtle structural plasticity of the SF in their ability to adapt to different ion types (Saponaro et al., 2021; Zhang et al., 2021).

It was already known that the SF is one of the structural elements responsible for gating in many pore loop channels. The balance between conductive, asymmetric, collapsed or disordered SF states is very important for the specific electrophysiological profile of a channel. The various constricted states of the SF, both collapsed and asymmetric, with a narrowed ion pore all show stabilization by either water molecules or a residue sidechain behind the SF. Interestingly, it seems that the constriction of the conduction pathway can be found at either the S2 or S0 ion binding site of the SF, where the constriction at the S0 site appears to be linked to an asymmetric state of the channel. It is unclear if all these different modes lead to the same behavior of C-type inactivation.

It is particularly challenging to obtain high-resolution structural information on the SF of ion channels by methods such as X-ray crystallography or cryo-EM when this is a dynamic region. MD simulations and NMR can provide additional information taking structural plasticity into account which aids the further understanding of ion channel functioning. An emerging theory links ion selectivity of the channel and SF disorder. It was first shown by us that multiple SF conformations are essential for non-selective ion conduction in NaK, with a symmetric state responsible for potassium and an asymmetric state responsible for sodium conduction (Shi et al., 2018). The disordered states of the SF, which might further influence the electrophysiology of the ion channel, could also play an important role in this. We expect that ssNMR in combination with MD simulations will continue to allow characterization of these dynamic SF states. More research is certainly needed to fully clarify the functional role of SF structural plasticity in pore loop channels.

## Author Contributions

KH wrote the original draft and prepared the figures. All authors contributed to the writing of the manuscript.

## Conflict of Interest

The authors declare that the research was conducted in the absence of any commercial or financial relationships that could be construed as a potential conflict of interest.

## Publisher’s Note

All claims expressed in this article are solely those of the authors and do not necessarily represent those of their affiliated organizations, or those of the publisher, the editors and the reviewers. Any product that may be evaluated in this article, or claim that may be made by its manufacturer, is not guaranteed or endorsed by the publisher.

## References

[B1] Alam A. Jiang Y. (2009). High-resolution structure of the open NaK channel. *Nat. Struct. Mol. Biol.* 16 30–34. 10.1038/nsmb.1531 19098917 PMC2615073

[B2] Bhate M. P. Wylie B. J. Tian L. Mcdermott A. E. (2010). Conformational dynamics in the selectivity filter of KcsA in response to potassium ion concentration. *J. Mol. Biol.* 401 155–166. 10.1016/j.jmb.2010.06.031 20600123 PMC2937177

[B3] Chakrapani S. Cordero-Morales J. F. Jogini V. Pan A. C. Cortes D. M. Roux B. (2011). On the structural basis of modal gating behavior in K+ channels. *Nat. Struct. Mol. Biol.* 18:67. 10.1038/nsmb.1968 21186363 PMC3059741

[B4] Cheng W. W. Mccoy J. G. Thompson A. N. Nichols C. G. Nimigean C. M. (2011). Mechanism for selectivity-inactivation coupling in KcsA potassium channels. *Proc. Natl. Acad. Sci. U. S. A.* 108 5272–5277. 10.1073/pnas.1014186108 21402935 PMC3069191

[B5] Chill J. H. Louis J. M. Miller C. Bax A. (2006). NMR study of the tetrameric KcsA potassium channel in detergent micelles. *Protein Sci.* 15 684–698. 10.1110/ps.051954706 16522799 PMC2242490

[B6] Cordero-Morales J. F. Cuello L. G. Zhao Y. Jogini V. Cortes D. M. Roux B. (2006). Molecular determinants of gating at the potassium-channel selectivity filter. *Nat. Struct. Mol. Biol.* 13 311–318. 10.1038/nsmb1069 16532009

[B7] Cordero-Morales J. F. Jogini V. Lewis A. Vasquez V. Cortes D. M. Roux B. (2007). Molecular driving forces determining potassium channel slow inactivation. *Nat. Struct. Mol. Biol.* 14, 1062–1069. 10.1038/nsmb1309 17922012

[B8] Cuello L. G. Jogini V. Cortes D. M. Pan A. C. Gagnon D. G. Dalmas O. (2010). Structural basis for the coupling between activation and inactivation gates in K(+) channels. *Nature* 466, 272–275. 10.1038/nature09136 20613845 PMC3033755

[B9] Doyle D. A. Morais Cabral J. Pfuetzner R. A. Kuo A. Gulbis J. M. Cohen S. L. (1998). The structure of the potassium channel: molecular basis of K+ conduction and selectivity. *Science* 280 69–77. 10.1126/science.280.5360.69 9525859

[B10] Heginbotham L. Lu Z. Abramson T. Mackinnon R. (1994). Mutations in the K+ channel signature sequence. *Biophys. J.* 66 1061–1067. 10.1016/S0006-3495(94)80887-2 8038378 PMC1275813

[B11] Hendriks K. Oster C. Shi C. Sun H. Lange A. (2021). Sodium ions do not stabilize the selectivity filter of a potassium channel. *J. Mol. Biol.* 433:167091. 10.1016/j.jmb.2021.167091 34090923

[B12] Jekhmane S. Medeiros-Silva J. Li J. Kummerer F. Muller-Hermes C. Baldus M. (2019). Shifts in the selectivity filter dynamics cause modal gating in K(+) channels. *Nat. Commun.* 10:123. 10.1038/s41467-018-07973-6 30631074 PMC6328603

[B13] Kim J. B. (2014). Channelopathies. *Korean J. Pediatr.* 57 1–18. 10.3345/kjp.2014.57.1.1 24578711 PMC3935107

[B14] Kopec W. Rothberg B. S. De Groot B. L. (2019). Molecular mechanism of a potassium channel gating through activation gate-selectivity filter coupling. *Nat. Commun.* 10:5366. 10.1038/s41467-019-13227-w 31772184 PMC6879586

[B15] Lee C. H. MacKinnon R. (2017). Structures of the Human HCN1 Hyperpolarization-Activated Channel. *Cell* 168:e111. 10.1016/j.cell.2016.12.023 28086084 PMC5496774

[B16] Lewis A. Kurauskas V. Tonelli M. Henzler-Wildman K. (2021). Ion-dependent structure, dynamics, and allosteric coupling in a non-selective cation channel. *Nat. Commun.* 12:6225. 10.1038/s41467-021-26538-8 34711838 PMC8553846

[B17] Li B. Rietmeijer R. A. Brohawn S. G. (2020). Structural basis for pH gating of the two-pore domain K(+) channel TASK2. *Nature* 586 457–462. 10.1038/s41586-020-2770-2 32999458 PMC8628578

[B18] Li J. Ostmeyer J. Cuello L. G. Perozo E. Roux B. (2018). Rapid constriction of the selectivity filter underlies C-type inactivation in the KcsA potassium channel. *J. Gen. Physiol.* 150 1408–1420. 10.1085/jgp.201812082 30072373 PMC6168234

[B19] Li J. Shen R. Reddy B. Perozo E. Roux B. (2021a). Mechanism of C-type inactivation in the hERG potassium channel. *Sci. Adv.* 7:eabd6203. 10.1126/sciadv.abd6203 33514547 PMC7846155

[B20] Li J. Shen R. Rohaim A. Mendoza Uriarte R. Fajer M. Perozo E. (2021b). Computational study of non-conductive selectivity filter conformations and C-type inactivation in a voltage-dependent potassium channel. *J. Gen. Physiol.* 153:e20211287. 10.1085/jgp.202112875 34357375 PMC8352720

[B21] Li M. Zhou X. Wang S. Michailidis I. Gong Y. Su D. (2017). Structure of a eukaryotic cyclic-nucleotide-gated channel. *Nature* 542 60–65. 10.1038/nature20819 28099415 PMC5783306

[B22] Liu S. Bian X. Lockless S. W. (2012). Preferential binding of K+ ions in the selectivity filter at equilibrium explains high selectivity of K+ channels. *J. Gen. Physiol.* 140 671–679. 10.1085/jgp.201210855 23148260 PMC3514730

[B23] Lolicato M. Natale A. M. Abderemane-Ali F. Crottes D. Capponi S. Duman R. (2020). K2P channel C-type gating involves asymmetric selectivity filter order-disorder transitions. *Sci. Adv.* 6:eabc9174. 10.1126/sciadv.abc9174 33127683 PMC7608817

[B24] MacKinnon R. (1995). Pore loops: an emerging theme in ion channel structure. *Neuron* 14 889–892. 10.1016/0896-6273(95)90327-5 7538310

[B25] Mandala V. S. Williams J. K. Hong M. (2018). Structure and Dynamics of Membrane Proteins from Solid-State NMR. *Annu. Rev. Biophys.* 47 201–222. 10.1146/annurev-biophys-070816-033712 29498890 PMC6312106

[B26] Miranda W. E. Demarco K. R. Guo J. Duff H. J. Vorobyov I. Clancy C. E. (2020). Selectivity filter modalities and rapid inactivation of the hERG1 channel. *Proc. Natl. Acad. Sci. U. S. A.* 117 2795–2804. 10.1073/pnas.1909196117 31980532 PMC7022143

[B27] Naylor C. E. Bagneris C. Decaen P. G. Sula A. Scaglione A. Clapham D. E. (2016). Molecular basis of ion permeability in a voltage-gated sodium channel. *EMBO J.* 35 820–830. 10.15252/embj.201593285 26873592 PMC4972137

[B28] Öster C. Hendriks K. Kopec W. Chevelkov V. Shi C. Michl D. (2019). The conduction pathway of potassium channels is water free under physiological conditions. *Sci. Adv.* 5:eaaw6756. 10.1126/sciadv.aaw6756 31392272 PMC6669007

[B29] Payandeh J. Scheuer T. Zheng N. Catterall W. A. (2011). The crystal structure of a voltage-gated sodium channel. *Nature* 475 353–358. 10.1038/nature10238 21743477 PMC3266868

[B30] Roy R. N. Hendriks K. Kopec W. Abdolvand S. Weiss K. L. De Groot B. L. (2021). Structural plasticity of the selectivity filter in a nonselective ion channel. *IUCrJ* 8 421–430. 10.1107/S205225252100213X 33953928 PMC8086165

[B31] Saponaro A. Bauer D. Giese M. H. Swuec P. Porro A. Gasparri F. (2021). Gating movements and ion permeation in HCN4 pacemaker channels. *Mol. Cell.* 81 2929–2943.e6. 10.1016/j.molcel.2021.05.033 34166608 PMC8294335

[B32] Sauer D. B. Zeng W. Raghunathan S. Jiang Y. (2011). Protein interactions central to stabilizing the K+ channel selectivity filter in a four-sited configuration for selective K+ permeation. *Proc. Natl. Acad. Sci. U. S. A.* 108 16634–16639. 10.1073/pnas.1111688108 21933962 PMC3189067

[B33] Schewe M. Nematian-Ardestani E. Sun H. Musinszki M. Cordeiro S. Bucci G. (2016). A Non-canonical Voltage-Sensing Mechanism Controls Gating in K2P K(+) Channels. *Cell* 164 937–949. 10.1016/j.cell.2016.02.002 26919430 PMC4771873

[B34] Shi C. He Y. Hendriks K. De Groot B. L. Cai X. Tian C. (2018). A single NaK channel conformation is not enough for non-selective ion conduction. *Nat. Commun.* 9:717. 10.1038/s41467-018-03179-y 29459730 PMC5818664

[B35] Shi N. Ye S. Alam A. Chen L. Jiang Y. (2006). Atomic structure of a Na+- and K+-conducting channel. *Nature* 440 570–574. 10.1038/s41467-018-03179-y 16467789

[B36] Van Der Cruijsen E. A. Prokofyev A. V. Pongs O. Baldus M. (2017). Probing Conformational Changes during the Gating Cycle of a Potassium Channel in Lipid Bilayers. *Biophys. J.* 112 99–108. 10.1016/j.bpj.2016.12.001 28076820 PMC5232892

[B37] Wang S. Lee S. J. Maksaev G. Fang X. Zuo C. Nichols C. G. (2019). Potassium channel selectivity filter dynamics revealed by single-molecule FRET. *Nat. Chem. Biol.* 15 377–383. 10.1038/s41589-019-0240-7 30833778 PMC6430689

[B38] Wang W. MacKinnon R. (2017). Cryo-EM Structure of the Open Human Ether-a-go-go-Related K(+) Channel hERG. *Cell* 169 422–430.e10. 10.1016/j.cell.2017.03.048 28431243 PMC5484391

[B39] Wylie B. J. Bhate M. P. Mcdermott A. E. (2014). Transmembrane allosteric coupling of the gates in a potassium channel. *Proc. Natl. Acad. Sci. U. S. A.* 111 185–190. 10.1073/pnas.1319577110 24344306 PMC3890889

[B40] Xu Y. Zhang D. Rogawski R. Nimigean C. M. Mcdermott A. E. (2019). Identifying coupled clusters of allostery participants through chemical shift perturbations. *Proc. Natl. Acad. Sci. U. S. A.* 116 2078–2085. 10.1073/pnas.1811168116 30679272 PMC6369819

[B41] Xue J. Han Y. Zeng W. Wang Y. Jiang Y. (2021). Structural mechanisms of gating and selectivity of human rod CNGA1 channel. *Neuron* 109 1302–1313.e04. 10.1016/j.neuron.2021.02.007 33651975 PMC8068614

[B42] Zhang K. Julius D. Cheng Y. (2021). Structural snapshots of TRPV1 reveal mechanism of polymodal functionality. *Cell* 185 5138–5150.e12. 10.1016/j.cell.2021.08.012 34496225 PMC8488022

[B43] Zhou Y. Morais-Cabral J. H. Kaufman A. Mackinnon R. (2001). Chemistry of ion coordination and hydration revealed by a K+ channel-Fab complex at 2.0 A resolution. *Nature* 414 43–48. 10.1038/35102009 11689936

